# On the Use of the Soda Tartarizata and Potassæ Sulphus in Diseased States of the Mucous Membrane of the Stomach and Bowels

**Published:** 1823-02

**Authors:** Charles Waller


ART. X.—*On the Use of the Soda Tartarizata and Potassæ Sulphus in diseased States of the Mucous Membrane of the Stomach and Bowels.* By CHARLES WALLER, Esq.

I HAVE been induced to send you, for insertion in your Journal, a few of the many cases I have witnessed, in which the employment of the soda tartarizata and the potassæ sulphas in diseased states of the mucous membrane of the stomach and bowels have proved beneficial, from a conviction that their powers as remedial agents are not sufficiently known, or, at least, are not duly appreciated by the profession at large. I do not mean to bring them forward as *new* remedies; nor did their use in these affections originate with myself, for I am indebted to a physician of great and deserved celebrity in this town for first directing my attention to the subject. In many cases, these salts have effected a cure, after the usual means for checking increased discharges from the bowels had failed: I allude to the Mist. cretæ cum opio,—a practice which, in my opinion, is combating the *effect*, whilst the *cause* is still suffered to remain.

CASE I. Mary Sparks, æt. 54.—*Symptoms.* Violent vomiting and purging; a single table-spoonful of gruel, if swallowed, was instantly ejected.—*Treatment.* Sodæ Tart. ℈ij. 4tis horis. —*Result.* The first dose was not retained on her stomach. The vomiting and purging, however, afterwards ceased; and in three days she was well.

CASE II. Jane Mansfield, æt. 30.—*Symptoms.* Great heat and pain at the stomach, with constant vomiting.—*Treatment.* Emp. Lyttæ scrob. cord. Sodæ Tart. ʒss. 3tis horis.—*Result.* By the next morning the vomiting had ceased, and did not return.

Case III. Mary Chambers, æt. 47.—*Symptoms.* Tenesmus, with great discharge of mucus and blood.—*Treatment.* Sodæ Tart. ʒj.; Sodæ Carb. gr. xv. 4tis horis.—*Result.* By continuing the medicine for three days, she completely recovered.

Case IV. Jane Collins, æt. 30.—*Symptoms.* Diarrhœa, with violent pains.—*Treatment.* Sodæ Tart. ʒss. 4tis horis.—*Result.* In less than twenty-four hours she was relieved; and, on the third day, not having had a motion, it was necessary to administer a more powerful laxative.

Case V. John Hexley, æt. 36. This man was under cure for colica pictonum, and was seized with diarrhœa, with discharge of blood, and a great degree of tenesmus.—*Treatment.* Pot. Sulph. ʒj. ter die.—*Result.* Bowels quite comfortable in four days.

Case VI. Harriet Wood, æt. 17.—*Symptoms.* Great tenesmus, with darting pains through the bowels, which are rather confined.—*Treatment.* Statim sumat Ol. Ricini, ℥j.; et postea Pot. Sulph. ʒj. 4tis horis.—*Result.* Perfectly relieved in twenty-four hours.

Case VII. Charlotte Dennis, æt. 22.—*Symptoms.* Great pain in the bowels, which are, however, regular.—*Treatment.* Potas. Sulph. ʒj. ter die.—*Result.* On the second day the pain was gone. The medicine had not caused any sensible effect.

Case VIII. Hannah Porser, æt. 21.—*Symptoms.* Pain in the bowels, with tenesmus, and great discharge of blood.—*Treatment.* Potas. Sulph. ʒj. ter die.—*Result.* In two days, the discharge of blood and the tenesmus were relieved. As she was much purged by the medicine, she was ordered it in less quantity.

Case IX. Mary Hexley, æt. 30, wife of J. H. (Case 5th.)—This was a much more serious case: she had severe pains in the bowels; great tenderness upon the application of any considerable pressure; she had most violent tenesmus, with great discharge of mucus and blood, the proper feculent matter being wholly retained; pulse feeble and quick; tongue coated with a dark brown fur. She had been ordered opium and port wine. I saw her on the 4th of November, and ordered her Potas. Sulp. ʒj. 4tis horis: her diet to consist of gruel and tea; the wine to be omitted.

*Nov. 5th.*—Has been much purged by the medicine: in fact, it operated so powerfully that she was in a state of alarming syncope for some time. This had gone off before I saw her, and she expressed herself to be much easier. Her stools are feculent, very little mucus, and no blood, being mixed with them. Tenesmus relieved; tongue much whiter.—Repetatur Pot. Sulph. ℨss. 4tis horis.

*6th.*—Her countenance, to-day, is much improved; tongue cleaner; pulse much calmer. She has frequent evacuations, which are feculent; complains of hemorrhoids. Has restless nights; no tenesmus. As I had succeeded in emptying the bowels of the feculent matter which had been locked up in them, I gave her Ext. Opii, gr. j. nocte, in addition to the Potas. Sulph. A little rice food was now allowed her.

*8th.*—Every symptom relieved; tongue clean, pulse natural; no tenesmus; has only one or two stools daily.

111, *Aldersgate-street; Nov.* 11*th*, 1822.

